# Millimeter-Wave-Based Spoof Localized Surface Plasmonic Resonator for Sensing Glucose Concentration

**DOI:** 10.3390/bios11100358

**Published:** 2021-09-28

**Authors:** Yelim Kim, Ahmed Salim, Sungjoon Lim

**Affiliations:** School of Electrical and Electronics Engineering, Chung-Ang University, Seoul 06974, Korea; kyelim6915@naver.com (Y.K.); ahmedsalim789@gmail.com (A.S.)

**Keywords:** spoof localized surface plasmon polariton, sensor, glucose solution, millimeter wave, metamaterial

## Abstract

Glucose-monitoring sensors are necessary and have been extensively studied to prevent and control health problems caused by diabetes. Spoof localized surface plasmon (LSP) resonance sensors have been investigated for chemical sensing and biosensing. A spoof LSP has similar characteristics to an LSP in the microwave or terahertz frequency range but with certain advantages, such as a high-quality factor and improved sensitivity. In general, microwave spoof LSP resonator-based glucose sensors have been studied. In this study, a millimeter-wave-based spoof surface plasmonic resonator sensor is designed to measure glucose concentrations. The millimeter-wave-based sensor has a smaller chip size and higher sensitivity than microwave-frequency sensors. Therefore, the microfluidic channel was designed to be reusable and able to operate with a small sample volume. For alignment, a polydimethylsiloxane channel was simultaneously fabricated using a multilayer bonding film to attach the upper side of the pattern, which is concentrated in the electromagnetic field. This real-time sensor detects the glucose concentration via changes in the S11 parameter and operates at 28 GHz with an average sensitivity of 0.015669 dB/(mg/dL) within the 0–300 mg/dL range. The minimum detectable concentration and the distinguishable signal are 1 mg/dL and 0.015669 dB, respectively, from a 3.4 μL sample. The reusability and reproducibility were assessed through replicates.

## 1. Introduction

Diabetes is a serious disease that currently affects the health of large populations worldwide and causes various health and lifestyle complications. Globally, the number of diabetic patients currently accounts for >8.5% of the adult population, and the prevalence has been steadily increasing. Diabetes is caused by inadequate absorption of glucose from the blood, mainly due to problems with insulin production. Hence, blood glucose concentration monitoring is necessary to prevent and control diabetes and related complications. Thus, technologies sensing blood glucose concentration are attracting much attention in the medical field. Sensing techniques, such as thermal, optical, mechanical, and microwave-based methods, have been proposed. Glucose sensors using any of these technologies should be small in size and be able to operate multiple times and on small samples, with high sensitivity, accuracy, and resolution [1,2,3,4,5,6].

In general, electrochemical or optical sensors are used for measuring glucose concentration. For example, electrochemical enzyme-based sensors involving finger pricking is widely used. Although there are few commercial microwave-based sensors, these sensors are attracting much attention due to their advantages, such as non-invasiveness, low cost, and easy fabrication. In microwave-based glucose sensors, an epsilon negative unit-cell resonator, complementary electric LC resonator, or passive components are used [7,8,9,10].

Localized surface plasmon (LSP) is defined as the confinement of a surface plasmon (SP) to nanoparticle size. SP refers to electromagnetic field (E-field) propagation along the interface between a metal and a dielectric material at the optical frequency, and LSP is the oscillations of free electrons on metallic surfaces. LSP-based methods are used in many applications, such as lenses, waveguides, and solar cells. In sensor applications, LSP sensors typically have high sensitivity because of their confined mode profiles and near-field enhancements [11,12,13]. In addition, various methods have been studied for optical LSP-based glucose sensing.

However, these methods are only used at the optical frequency. The spoof LSP-based sensor has also been studied because of its merits, such as a high-quality factor (Q-factor) and high sensitivity. This method has been developed from periodic holes made by ultrathin corrugated metallic disks. In addition, corrugated spoof LSP resonators have been developed in several shapes to improve the Q-factor. Therefore, we designed a millimeter-wave-based spoof LSP resonator sensor to achieve small physical size and sample volume, as well as a high Q-factor and sensitivity level. The structure used to construct these spoof LSPs is called the plasmonic metamaterial. The metamaterial is an artificial material that has negative indices of refraction and is generally used in applications such as antennas, absorbers, and lenses [14,15,16,17,18,19,20]. In general, the corrugated structure used in spoof LSP resonator-based sensors operates at the microwave frequency.

In this study, a spoof LSP resonator was used to design a glucose sensor that operates at millimeter-wave frequency. Millimeter-wave-based sensors have the merits of high data transmission rates for communication, enhanced security, and reduced interference while supporting miniaturized sensor sizes [21,22]. In addition, this sensor uses a microfluidic channel fabricated from polydimethylsiloxane (PDMS), and channels are connected by using a multilayer bonding film. The designed PDMS channel reduces the sample volume and increases the detection sensitivity. The channel through which the glucose solution is injected is considered the loading position, where the E-field of the sensor is concentrated. Further, the channel structure reduces the effects of air bubbles. The multilayer bonding film reduces any remaining sample noise, and our design confers a high Q-factor to the spoof LSP resonator. Accordingly, the sensor presented here has a small size, high Q-factor, and high sensitivity, and can operate on small volumes of samples.

We fabricated sensors by using either of two different designs of microfluidic channels. The resulting sensors could detect concentrations in the range of 0–300 mg/dL. The channels were designed and measured nine samples to assess the sensing performance of the sensor. The sensors detect differences in glucose concentration according to changes in the reflection coefficients; the magnitude of the reflection coefficient increases with the glucose concentration. In addition, sensor sensitivity, reusability, and reproducibility were evaluated. The average sensitivity was 0.015669 dB with a 3.4 μL sample volume, and the sensors could be used up to 60 times. Accordingly, their reproducibility was approximately 0.3%. The results obtained using the PDMS channel were compared with the results obtained from sensors based on spoof SPs or LSPs as well as other state-of-the-art glucose sensors. The proposed sensor was observed to have high sensitivity for a miniaturized device that can operate on small volumes of samples. The sensor also has a low detection limit of 1 mg/dL.

## 2. Materials and Methods

### 2.1. Preparation of the Materials and Glucose-Solution Sample

The PDMS and bonding film used to fabricate the microfluidic channels were manufactured by Shielding Solutions Ltd., Braintree, UK, and Adhesives Research, Glen Rock, PA, USA, respectively. D-(+)-Glucose powder and deionized (DI) water (pH 6.4) were purchased from Sigma Aldrich. The DI water was produced via reversed osmosis. Glucose-solution samples were prepared in-house via mixing at 40–45 kHz.

### 2.2. Complex Permittivity of the Solution Samples

**Figure 1:**
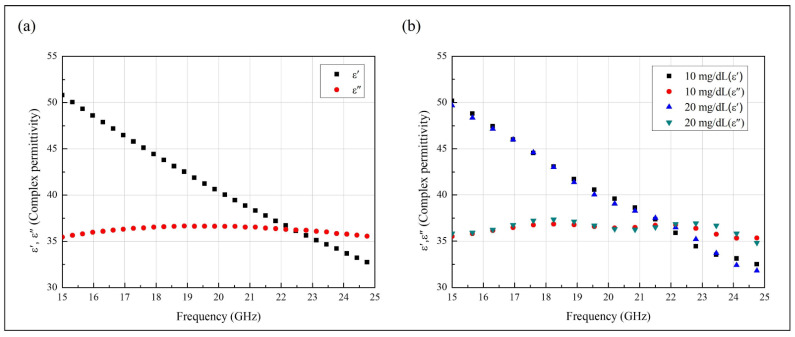
Complex relative permittivity ε′ and ε″ of (**a**) deionized water and (**b**) 10 and 20 mg/dL glucose, all measured at 27.8 °C.

DI water and glucose solutions were prepared to investigate the detection performances of the designed glucose sensors. For the measurements, glucose-solution samples with concentrations of 0–300 mg/dL were prepared. The complex relative permittivity was measured using DI water and 10 and 20 mg/dL glucose, and the temperature of the liquid was maintained at 27.8 °C. Figure 1 shows the measured complex permittivity of the DI water and glucose solutions from 15 to 25 GHz. The proposed sensor has an operating frequency range up to 40 GHz; however, owing to the accuracy limits of the machine, permittivity was measured only until 25 GHz. To measure these electromagnetic properties of the sample solutions, we used Keysight N1501A and 8510C equipment. These properties can also be measured via various techniques, such as using a resonator, a coaxial probe, and a cavity. ε′ and ε″ are the real and imaginary parts of the complex relative permittivity. Figure 1a shows the measured complex relative permittivity of the DI water. The real part ε′ decreased from 50.7871 to 32.4382, and the imaginary part ε″ increased from 35.4508 to approximately 20 GHz and then decreased to 35.4901.

Figure 1b shows the measured permittivity of 10 and 20 mg/dL glucose. With a 10 mg/dL concentration, ε′ decreased from 50.1766 to 31.7046, and ε″ increased from 35.5241 to approximately 20 GHz and decreased thereafter to 35.1749. With a 20 mg/dL concentration, ε′ decreased from 49.6697 to 31.292, and ε″ increased from 35.8409 to approximately 20 GHz and decreased thereafter to 36.8368. When the glucose concentration was increased by 10 mg/dL, the average values of ε′ and ε″ increased by 0.49 and decreased by 0.13, respectively.

The complex relative permittivity and loss tangent were defined by the measured values of ε′ and ε″. Equation (1) represents the complex relative permittivity $\varepsilon_{c}$:

(1) $$\varepsilon_{c}=\;\varepsilon \text{′}+j\varepsilon \prime \prime .$$

Further, the tangent loss tanδ can be defined as the ratio of the real part to the imaginary part of the complex relative permittivity, as follows:

(2) $$tan\delta =\frac{\varepsilon \text{′}}{\varepsilon \prime \prime }.$$

These results show the dielectric properties of the prepared DI water and glucose-solution samples. These dielectric properties depend on the frequency used. The values calculated using Equations (1) and (2) represent the changes in the glucose concentration, which affect the dielectric constant and loss tangent. With increases in the glucose concentration, the dielectric constant decreases, while the loss tangent increases. Moreover, the temperature changes also affect the dielectric constant and loss tangent of glucose solution. Thus, the samples were maintained at a constant temperature during the measurements for accuracy [23,24,25,26].

### 2.3. Sensor Design Based on the Spoof LSP Resonator

**Figure 2:**
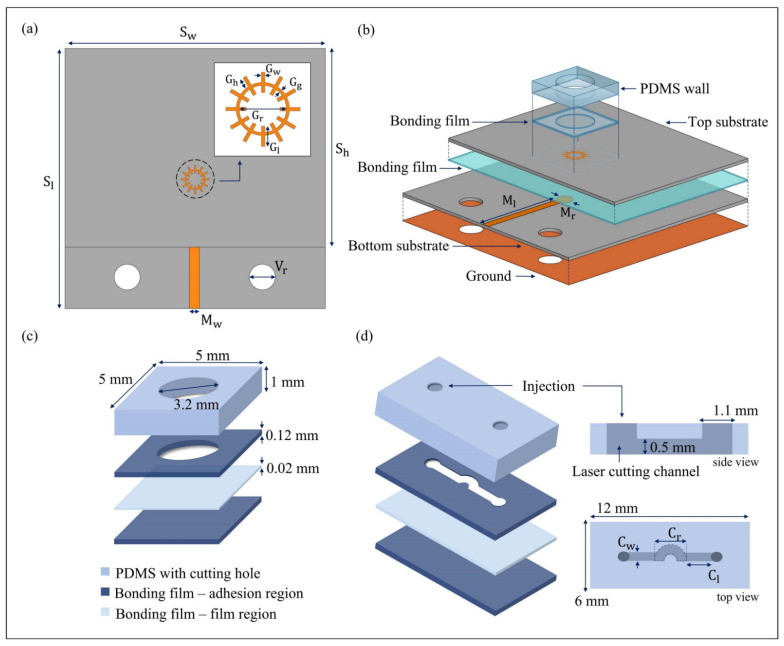
Schematic of the proposed sensor and PDMS channel. (**a**) Top view of the sensor and magnified design of the spoof LSP resonator. (**b**) Layer design of the sensor with microfluidic channels and their alignments. (**c**) PDMS wall and bonding film layer. (**d**) Detailed PDMS-channel design showing the bonding film layer.

Figure 2 shows the schematic of the proposed sensor and microfluidic channels with a multilayer bonding film. The proposed sensor consists of two layers and each layer is fabricated using the same Rogers Duroid 5880 substrate. The thickness of the substrate is 0.25 mm and that of the attached copper is 0.018 mm; the dielectric constant $\varepsilon_{r}$ and loss tangent tanδ of the substrate are 2.2 and 0.0009, respectively. Figure 2a shows the top view of the designed sensor, with the parameters of the top layer pattern. The top substrate has no ground and only the top pattern. The length from the center of the spoof LSP resonator pattern to the edge of the substrate is 4.9 mm. The pattern has a ring-shaped resonator with periodic grooves and a higher Q-factor than the conventional spoof LSP resonator. This modified resonator has the merits of restraining high-order modes and enhancing the fundamental mode. The width $S_{w}$ and length $S_{h}$ of the top substrate are 20 and 14.9 mm, respectively. The diameter $G_{r}$ and width $G_{w}$ of the interior of the ring are 1.4 and 0.1 mm, respectively. The parameter $V_{r}$ of the bottom substrate is the hole for connecting the K-band connectors and has a diameter of 1.98 mm. The bottom substrate consists of the ground and a 50 Ω microstrip line. The microstrip line has a circular end for reducing the reflected waves. The circular shape increases the efficiency of the transfer of the E-field to the pattern. The bottom substrate has the same width as the top substrate but a different length $S_{l}$ (20 mm) [27]. Figure 2b shows the 3D view of each layer of the sensor, locations of the bonding films, and the microfluidic channel. The width of the microstrip line, $M_{w}$, is 0.7 mm and the length from the center of the circle $M_{l}$ is 8.8 mm; the diameter of the circle, $M_{r}$, is 1.4 mm. The bonding film connects the top and bottom substrates and has the same size as the top substrate. The bonding film between the substrates is only a 0.12 mm-thick single adhesion layer. The PDMS channels are arranged at the center of the pattern; in the sensor design, the ground, microstrip line, and pattern were fabricated using copper, which has an electrical conductivity of $\sigma =5.8\times 10^{-7}\;{\mathrm{Sm}}^{-1}$.

Figure 2c,d shows the microfluidic channels made of PDMS and the bonding film layers constructed via the laser-cutting fabrication method. The thickness of the PDMS is 1 mm, and the bonding film is 0.12 mm thick at the adhesion region and 0.02 mm thick at the film region. The dielectric constant $\varepsilon_{r}$ and loss tangent tanδ of the PDMS are 2.7 and 0.035, respectively. The adhesion and film regions have $\varepsilon_{r}$ values of 2.35 and 2, respectively, with the same tanδ value of 0.002. Both the PDMS walls and channels were constructed using the same fabrication technique. The multilayer bonding film is composed of three layers, and the first adhesion layer with the same structure as the microfluidic channel is produced simultaneously with the PDMS using a laser. The bonding film, which consists of film and adhesion layers attached to the PDMS, was cut using a laser, and only the film layer was removed. Then, the film layer was attached to the side of the adhesion region of PDMS. This fabrication method was considered to prevent the misalignment between the channel and the bonding film and the remaining solution. When the bonding film is misaligned, the solution may leak, and small liquid bubbles can attach to the corners. Removal of the remaining sample solution is therefore necessary for accurate results. In addition, the middle layer film region allows clearer solution sample removal compared to the adhesion layer. Because the film region has less surface roughness and less adhesion to liquids than the conventional single-layer bonding film, which has an adhesion region, less sample remains in this method than the conventional method. Further, due to the high sensitivity of the sensor, the results are affected by the alignment of the microfluidic channel with the pattern. Alignment lines marked on the top and bottom layers are therefore used to reduce such positioning problems.

Figure 2c shows the design of the PDMS wall, which is 5 × 5 × 1 mm in size. The diameter of the wall is 3.2 mm; hence, the inside volume of the wall is 8.04 mm^3^. Thus, an 8 μL glucose solution is injected using a micropipette. Figure 2d shows the design of the PDMS channel and detailed parameters. The channel width and length are 12 and 6 mm, respectively. The diameter of the injection hole is 1.1 mm, and the internal channel thickness is 0.5 mm. The width of the channel, $C_{w}$, is 0.8 mm, including the curved side. The channel length from the edge of the curved outside diameter to the surface of the injection hole, $C_{l}$, is 1.6 mm. The diameter of the curved channel area, $C_{r}$, is 2.4 mm.

**Figure 3:**
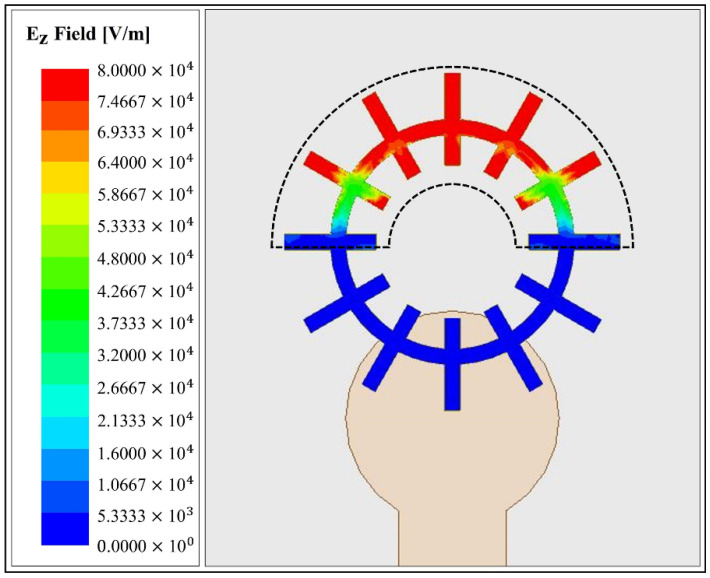
Simulated magnitudes of the E_z_ vectors of the E-field at a resonant frequency of 37.02 GHz.

The PDMS channel is designed to increase sensitivity. Figure 3 shows the simulated E-field concentration of the sensor at the resonant frequency of 37.02 GHz. The figure shows the magnitude of the E-field vector in the +z direction. The E_z_ vector fields are concentrated on the upper parts of the ring and grooves. The equivalent circuit of the spoof LSP has two parallel capacitances between each groove and is connected in series. In the equivalent circuit, when the capacitors are connected in parallel, the sensitivity of the loaded material is not affected by the loading position. However, in the case of series capacitance, the loading position is important for sensitivity. The series-connected capacitors have higher capacitance shifts when the sample is loaded only in the strong capacitance area. Thus, the sensitivity is higher in the capacitance-concentrated region than in the weak capacitance region. The area with the dotted lines refers to the strong capacitance concentration of the curved PDMS channel, designed specifically for increased sensitivity. Hence, only a 3.4 μL sample needs to be injected for measurement [28,29]. The reflected coefficients were used to assess the performance of the fabricated sensor. In the simulation, the resonant frequency and reflection coefficient of the designed sensor shifted in parallel to the changes in sample concentration. However, during the measurements, only the reflection coefficients were used because of the noise.

## 3. Results and Discussion

### 3.1. Measurement Results

**Figure 4:**
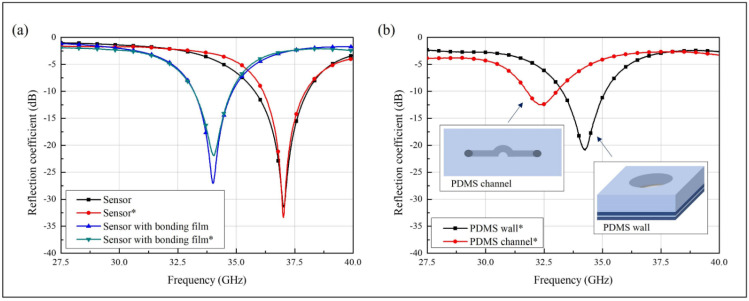
Simulated and measured results of (**a**) the sensor without or with the bonding films. (**b**) Measured results when the PDMS microfluidic channels were used (* is the measured result).

The fabricated glucose concentration sensor was evaluated using a vector network analyzer (VNA) with a 2.92 mm K-band connector. Figure 4 shows the simulated and measured results of the sensor. Figure 4a shows the simulated and measured values of the sensor without and with the bonding film. The measured value of the sensor has a resonant frequency of 37.02 GHz and a reflection coefficient of −33.38 dB. The calculated Q-factor of the measured sensor is 308.5. The measured results of the sensor with the bonding film have a 34.025 GHz resonant frequency and a −21.95 dB reflection coefficient. The calculated loaded Q-factor of the measured sensor with the bonding film is 87.3. The PDMS-channel designs used for the simulation and measurement with the bonding film are shown in Figure 2d. The simulated results of the sensor without the bonding film are similar to those with the bonding film. The simulated sensor has a resonant frequency of 34.035 GHz and a reflection coefficient of −31.47 dB. The calculated loaded Q-factor of the simulated sensor is 200.2. The simulated sensor with the bonding film has a 34.005 GHz resonant frequency and −27.07 dB reflection coefficient, with a Q-factor of 154.59. These differences in the resonant frequency can occur because of fabrication errors, such as in the groove widths and lengths. The Q-factor is calculated using Equation (3).

(3) $$\frac{f_{r}}{f_{3dB\;\left(upper\right)}-f_{3dB\;\left(lower\right)}}.$$

In Equation (3), $f_{r}$ is the resonant frequency, and $f_{3dB\;\left(upper\right)}$ and $f_{3dB\;\left(lower\right)}$ are the higher and lower frequencies compared to the resonant frequency, which are 3 dB different from the resonant frequency. Thus, the denominator is the 3 dB bandwidth. The calculated Q-factor shows that the spoof LSP-resonator-based sensor has a high Q-factor, and the used ring pattern located at the center of the grooves has a higher Q-factor than that of the original spoof LSP resonator design with periodic grooves located at the interface of the circle [30].

Figure 4b shows the measured sensor results for two different microfluidic-channel shapes. The PDMS wall and channel are attached to the sensor through three-layer bonding films of the same size as each PDMS. The sensor with PDMS channels has a resonant frequency of 32.325 GHz and a reflection coefficient of −12.48 dB, and the sensor with the PDMS wall has a 34.255 GHz resonant frequency and a −20.9 dB reflection coefficient. The measured results show the tendencies of the resonant frequency and reflection coefficient shifts.

### 3.2. Sensitivity of the Proposed Glucose Sensor

**Figure 5:**
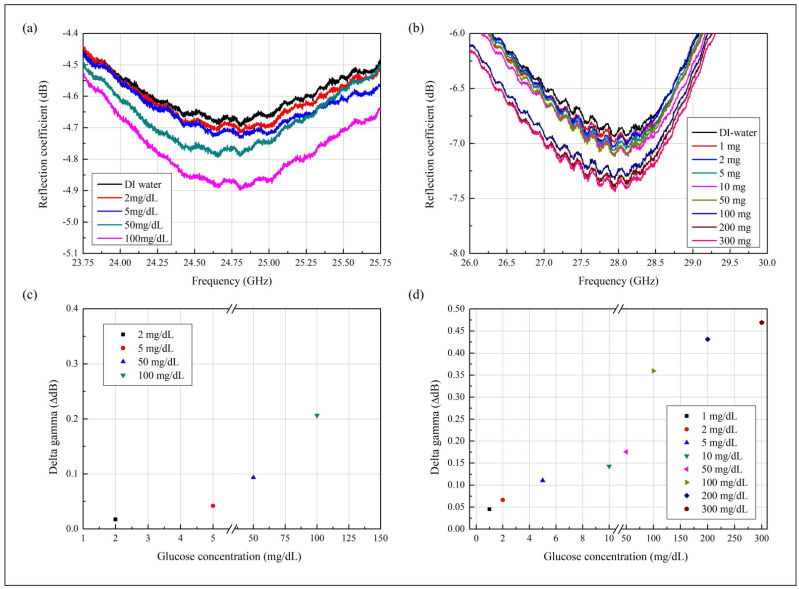
Measured reflection coefficients for the sensor with the (**a**) PDMS wall or (**b**) PDMS channel. Calculated delta gamma of the reflection coefficients for the sensor with the (**c**) PDMS wall at 24.8 GHz and 27.8 °C or (**d**) the PDMS channel at 28 GHz at 22.3 °C.

The sensitivity of the sensor with the PDMS wall was evaluated using 1, 2, 5, 10, 50, and 100 mg/dL glucose solutions at 27.8 °C, which is the temperature at which the relative complex permittivities were measured. Likewise, the sensitivity of the sensor with the PDMS channel was evaluated using 1, 2, 5, 10, 50, 100, 200, and 300 mg/dL samples at 22.3 °C. Figure 5 shows the measured results of the sensor with the microfluidic channels filled with DI water or glucose-solution samples. In Figure 5a, the measured reflection coefficient differences depend on the injected samples (DI water and glucose solutions of 1, 2, 5, 50, and 100 mg/dL). The resonant frequency of the sensor with the PDMS wall filled with DI water was 24.75 GHz. For these measurements, an 8 μL sample solution was injected and extruded using a micropipette. The 1 mg/dL glucose solution yielded the same result as the 2 mg/dL sample. Thus, the sensor with the PDMS wall can distinguish a difference with a maximum of 2 mg/dL.

The reflection coefficient at 24.8 GHz was then used to confirm the sensing performance for glucose. The reflection coefficient change delta gamma has the largest value at 24.8 GHz. Figure 5b shows that the measured reflection coefficients of the sensor with the PDMS channel depend on the glucose concentration. The results shown correspond to DI water and 1, 2, 5, 10, 50, 100, 200, and 300 mg/dL glucose. The resonant frequency of the sensor with the PDMS channel filled with DI water was 28 GHz; hence, 28 GHz was used to assess the sensing performance with changes in glucose concentration. The graphs show that the sensor with the PDMS channel can distinguish a difference of 1 mg/dL in glucose concentration. Results from the PDMS wall and channel both show that when the glucose concentration increases, the amplitude of the reflection coefficient also increases.

Figure 5c,d shows the calculated sensing performances as the differences between the measured reflection coefficients. Figure 5c shows the delta gamma calculated using the reflection coefficients at 24.8 GHz. At 24.8 GHz, the sensor with the PMDS wall exhibited reflection coefficients from −4.68 to −4.89 with the glucose concentration increasing from 0 (DI water) to 100 mg/dL. More specifically, the measured reflection coefficients were −4.69, −4.72, −4.78, and −4.89 dB at 2, 5, 50, and 100 mg/dL, respectively. The delta gamma values were calculated according to the results obtained using DI water. The calculated values have differences of 0.01729, 0.045215, 0.09342, and 0.20637 dB at 2, 5, 50, and 100 mg/dL, respectively, based on the dB value of DI water. Thus, the sensor with the PDMS wall has an average sensitivity of 525.17 dB/g/mL and a minimum detection limit of 2 mg/dL.

Figure 5d shows the assessment of the sensitivity by using the same method as in Figure 5c. The graph shows the calculated delta gamma values for the glucose concentrations. The results represent the calculated delta gamma values of the sensor with the PDMS channel at 28 GHz. The measured reflection coefficient of DI water was −6.89447 dB, and the values were −6.93943, −6.96078, −7.00517, −7.03714, −7.07025, −7.25414, −7.32565, and −7.36336 dB at 1, 2, 5, 10, 50, 100, 200, and 300 mg/dL, respectively. Thus, the calculated delta gamma values were 0.04496, 0.06631, 0.1107, 0.14267, 0.17578, 0.35967, 0.43118, and 0.46889 dB at 1, 2, 5, 10, 50, 100, 200, and 300 mg/dL according to the results obtained using DI water, respectively. The average sensitivity is 1566.9 dB/(g/mL), and the minimum detection limit is 1 mg/dL. These results show that the sensor can detect glucose concentrations by reflection coefficient shifts. The marked dotted lines shown in Figure 5c,d represent the linear ratio between delta gamma and glucose concentration. The inclination of the dot line shows the sensor with the PDMS channel has higher sensitivity than the sensor with the PDMS wall.

To investigate the sensor with either microfluidic channel, we used Equation (4).

(4) $$Sensitivity=\frac{∆dB}{∆c}=\frac{\left|dB_{c1}-dB_{c2}\right|}{\left|c_{1}-c_{2}\right|}.$$

The parameter $c_{n}$ is glucose concentration (mg/dL), and $dB_{cn}$ is the reflection coefficient of $c_{n}$ at the resonant frequency of each result. The sensitivities are determined according to the reflection coefficients and sample concentration. The results show that the proposed sensor has an average sensitivity of 0.01567 dB/(mg/dL).

### 3.3. Analytical Characterization of the Sensor

**Figure 6:**
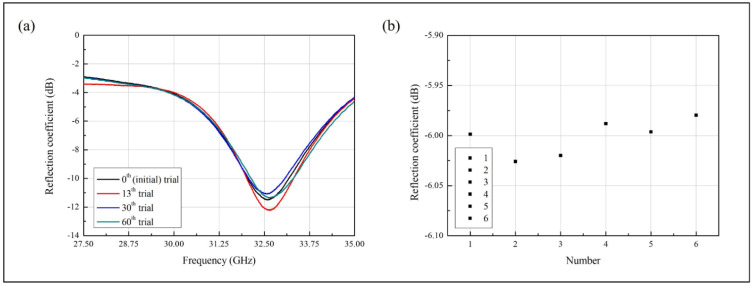
Measured reflection coefficients to investigate (**a**) the reusability and (**b**) the reproducibility of the sensor.

To investigate the analytical characteristics of the sensor, we experimented and calculated the reusability, reproducibility, and response time. The PDMS channel is a highly reusable material. It is flexible and can be fabricated in various designs. In Figure 6a, the fabricated sensor has a different resonant frequency at the 60th iteration of removing the injected sample. Until the 60th trial, the measured results have the same resonant frequency and only have a different reflection coefficient. During the measurement, the PDMS channel, which is filled with air, can be affected by environmental factors, such as vibrations. Therefore, to measure the reusability, we investigated the changes in resonant frequency.

Figure 6b shows the reproducibility of the sensor. The 300 mg/dL concentration sample was used for measurement, and due to the difficulty of maintaining the temperature, it was measured at 25.2 ℃. The average reflection coefficient was measured at −6.001431 dB, and the maximum and minimum values were −5.97966 and −6.02592 dB, respectively. The calculated average sensitivity was 0.01567 dB/(mg/dL), and the maximum and minimum values can be represented by 1.5 times the average 1 mg/dL sensitivity. Therefore, the sensor reproducibility relative standard deviation (RSD) is 0.3%. The RSD was calculated by the following Equation:

(5) $$\mathrm{RSD}=\frac{\mathrm{standard}\;\mathrm{deviration}}{\mathrm{average}}\times 100\%.$$

During the measurement, we used the VNA and VNA-measured S-parameters in real-time. The conventional frequency-based glucose sensors, such as those based on the microwave or millimeter wave frequency, can monitor biological reactions in real-time. Therefore, real-time monitoring is one of the advantages of using the microwave sensing technology method [31].

### 3.4. Performance Comparison of the Sensors

To investigate the sensing performance, we compared the designed glucose sensors with the conventional sensors based on spoof SP or LSP, as well as other state-of-the-art glucose sensors, by using multiple parameters important for a sensor, such as physical size, sample volumes, minimum distinguishable concentration, and sensitivity. Table 1 shows the comparison with the sensors based on spoof SP or LSP, including those used to sense chemicals other than glucose. The designed glucose sensors are smaller, have higher Q-factors, and can measure smaller-sample volumes than the conventional spoof LSP sensors. The sizes of the designed millimeter-wave-based sensors are similar to those of the sensors based on the quarter-mode LSP. The fabricated sensor also has better sensitivity and a higher Q-factor compared to the quarter-mode spoof LSP sensor, which operates at microwave frequency. This observation indicates that the sensors based on millimeter-wave frequency show better performance than the LSP sensors. The former sensors are smaller, can assess smaller sample volumes, have higher sensitivity than the latter, and do not require additional circuit parts. Moreover, conventional spoof SP- or LSP-based sensors have higher sensitivity to permittivity than sensors based on other methods because of the near-field enhancement [32,33,34,35].

In Table 2, the designed sensor is compared with other state-of-the-art glucose sensors. Other sensing techniques are also presented, such as coplanar waveguides (CPWs) with interdigital (IDT) structures and electric LC (ELCS) resonators, and complementary split-ring resonators (CSRRs). These sensors have miniaturized sizes despite operating at microwave frequencies. However, the required sample volume for sensing the glucose solution is different. For sensing the glucose concentration, these sensors need at least 5 times the sample volume needed in the proposed sensor. Thus, the proposed sensor has higher sensitivity and requires less sample volume than the other sensors [36,37,38,39,40,41]. However, microwave-based sensors have a limitation in selectivity because their detection is based on changes in the dielectric constant and loss tangent. For instance, when different mixed solutions have a similar dielectric constant and loss tangent, their frequency responses are similar so the target substance cannot be detected [42,43].

**Figure 7:**
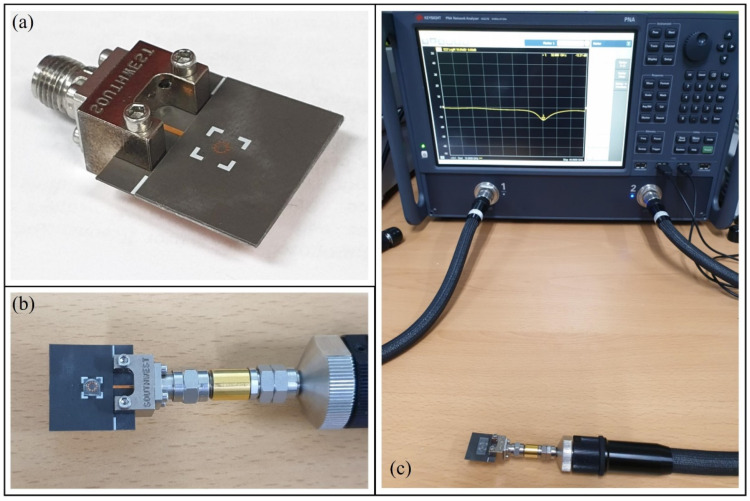
Photos of the fabricated sensor with the (**a**) connector and (**b**) PDMS wall filled with the glucose solution. (**c**) Measurement settings of the sensor with the PDMS channel.

Figure 7 shows the photos of the fabricated sensors and their measurement settings. Figure 7a shows the sensor with the top and bottom substrates attached using bonding films and connected to a 2.92 mm K-band connector. The white lines denote the position where the top substrate aligns with the PDMS wall. Figure 7b shows the top view of the sensor with the PDMS wall containing the glucose solution. Figure 7c shows the measurement settings using the VNA for the sensor with the PDMS channel.

## 4. Conclusions

In this study, we designed a spoof LSP resonator sensor that operates at millimeter-wave frequencies for sensing glucose concentrations. By measuring the complex relative permittivity of glucose solutions at various concentrations, we confirmed the dielectric properties of DI water and glucose solution. Glucose solutions in the range of 0−300 mg/dL were used for sensing, with a sample volume of 3.4 µL to investigate the sensor performance. The proposed sensor senses differences in glucose concentrations according to the changes in the reflection coefficients. The sensor using the PDMS channel with the multilayer bonding film has a sensitivity of 1566.9 dB/(g/mL), and it can distinguish a difference of 1 mg/dL. In addition, it can be reused 60 times and has a reproducibility of 0.3%. The proposed sensor was compared with other spoof LSP sensors and those using the microwave method. From these evaluations, it was observed that the proposed sensor has the advantages of being small, having high sensitivity, and the ability to work on small volumes of sample.

## Figures and Tables

**Table 1:** Comparison with sensors using spoof SP or LSP.

Title [Ref. No]	Operating Frequency (GHz)	Physical Size (mm^2^)	Sample Volume (𝛍L)	Q-Factor ^1^	Distinguishable Concentration (mg/dL)	Sensitivity	
						(dB/(g/mL))	(MHz/(g/mL))
This work	28	20 × 20	3.4	308.5	1	1566.9 ^2^	N/A
[15]	3.16−3.76 ^3^	20 ^4^ × 20 ^4^	101.7	N/A	1164	N/A	773
[32]	6.86−7.8	17 × 17	3.9	25	N/A	940 MHz shift (10 to 90% ethanol)	
[33]	6.67	34 × 34	6 & 15.5	N/A	N/A	detection two chemicals	
[34]	1.5−2.5	42 × 40	12	40,000	9	N/A	29,111.1
[35]	8−12	52 × 24	N/A	N/A	25	N/A	200,000

^1^ Measured value with only the sensor without any additional parts. ^2^ Calculated average value. ^3^ The value estimated from the plot. ^4^ Pattern size only.

**Table 2:** Comparison with state-of-the-art glucose sensors.

Title [Ref. No]	Sensing Technique	Operating Frequency (GHz)	Physical Size (mm^2^)	Sample Volume (𝛍L)	Q-Factor	Sensitivity	
						(dB/(g/mL))	(MHz/(g/mL))
This work	Spoof LSP resonance	28	20 × 20	3.4	308.5	1566.9	N/A
[23]	CSRR	2.9	26 × 40	N/A	N/A	7.5	N/A
[36]	CPW with IDT	3.9−4.12 ^2^	25.4 × 30	15	20 ^1^	15.3	235.32
[37]	CPW with ELC	3.41	16 ^3^ × 16 ^3^	20	N/A	3.73	N/A
[38]	CSRR driven by ISM radar	2.45	20 × 66	600	60 ^1^	N/A	125,000
[39]	Hilbert curve	6	20.4 × 40.4	500	62	1560	N/A
[40]	Microstrip	5.5−6.7	80 × 80	14,000	81 ^1^	N/A	54,000
[41]	WGM ^4^	50−70	50 × 7.64 ^3^	50–370	N/A	1000	N/A

^1^ Estimated value from the plot. ^2^ dB parameter used frequency. ^3^ Only pattern size. ^4^ Whispering gallery modes.

## Data Availability

The data presented in this study are available on request from the corresponding author.
